# Artificial-Intelligence-Based Clinical Decision Support Systems in Primary Care: A Scoping Review of Current Clinical Implementations

**DOI:** 10.3390/ejihpe14030045

**Published:** 2024-03-13

**Authors:** Cesar A. Gomez-Cabello, Sahar Borna, Sophia Pressman, Syed Ali Haider, Clifton R. Haider, Antonio J. Forte

**Affiliations:** 1 Division of Plastic Surgery, Mayo Clinic, Jacksonville, FL 32224, USA; 2 Department of Physiology and Biomedical Engineering, Mayo Clinic, Rochester, MN 55902, USA

**Keywords:** artificial intelligence, machine learning, clinical decision support systems, primary healthcare

## Abstract

Primary Care Physicians (PCPs) are the first point of contact in healthcare. Because PCPs face the challenge of managing diverse patient populations while maintaining up-to-date medical knowledge and updated health records, this study explores the current outcomes and effectiveness of implementing Artificial Intelligence-based Clinical Decision Support Systems (AI-CDSSs) in Primary Healthcare (PHC). Following the PRISMA-ScR guidelines, we systematically searched five databases, PubMed, Scopus, CINAHL, IEEE, and Google Scholar, and manually searched related articles. Only CDSSs powered by AI targeted to physicians and tested in real clinical PHC settings were included. From a total of 421 articles, 6 met our criteria. We found AI-CDSSs from the US, Netherlands, Spain, and China whose primary tasks included diagnosis support, management and treatment recommendations, and complication prediction. Secondary objectives included lessening physician work burden and reducing healthcare costs. While promising, the outcomes were hindered by physicians’ perceptions and cultural settings. This study underscores the potential of AI-CDSSs in improving clinical management, patient satisfaction, and safety while reducing physician workload. However, further work is needed to explore the broad spectrum of applications that the new AI-CDSSs have in several PHC real clinical settings and measure their clinical outcomes.

## 1. Introduction

Primary care stands as a cornerstone in healthcare, serving as the first point of contact and managing the most significant number of patients in the United States [1] and worldwide. It offers patient-centered, comprehensive, longitudinal, and coordinated care across settings [2]. Managing a large, heterogeneous population is a challenging task for Primary Care Physicians (PCPs), especially when many patients have concurrent chronic diseases and polypharmacy [3]. Keeping a complete health record and clinical knowledge up to date is essential. 

In 2007, the US government encouraged the introduction of Clinical Decision Support Systems (CDSSs) into Electronic Health Records (EHR), and by 2017, 40.2% of US hospitals had advanced CDSS capabilities [4]. CDSSs aid physicians in diagnosis, disease management, prescription, and drug control, often through alarm systems [5,6]. They have been especially effective in increasing adherence to clinical guidelines, applying prevention and public health strategies, and improving patient safety [3,7]. Furthermore, with CDSSs’ integration into EHRs, the incidence of pharmacological adverse events has decreased, and both recommendations and alerts have become personalized [7,8]. 

According to a meta-analysis, CDSSs improved the average percentage of patients receiving the desired care element by 5.8% [9]. Even with CDSSs supporting PCPs in making up-to-date clinical decisions [10], their impact on morbidity and mortality in Primary Healthcare (PHC) has not been conclusively demonstrated [11]. Moreover, PCPs may face difficulties co-managing patients with specialties due to discrepancies in the recommendations given by the specialists and their CDSSs or outdated EHRs [11].

Although the concept of Artificial Intelligence (AI) was first introduced seven decades ago, its evolution began in 2010 with the enhancement of graphic processing units [12,13,14,15]. AI can mimic human reasoning and behavior [12,13,15,16] and handle the increasing volume of medical data within healthcare systems [17]. Machine learning (ML) is the most common AI technique used, and it can be categorized into three types: supervised, unsupervised, and reinforcement algorithms [12,18]. Massive training datasets are used as input to train ML algorithms to make accurate predictions, allowing computers to learn without explicit programming [12,15,16,19]. With deep learning (DL), a subset of ML, programs can learn and modify themselves by feedback from multiple layers, achieving the most stable prediction outcome [12,14,16,19].

In contrast to knowledge-based CDSSs with if–then rules, non-knowledge-based CDSSs leverage AI, improving their diagnostic, prognostic, and administrative capabilities [5,20]. These models can potentially reduce medical errors while increasing physician efficiency and productivity [21], allowing them to focus on tasks that require unique human skills, such as attending to individual patient concerns [22]. 

Despite substantial efforts to evaluate CDSSs’ effectiveness across various medical specialties, to our knowledge, there are just two reviews concerning PHC [4,11]. The same can be said about AI, which primarily has applications in oncology, pulmonology, cardiovascular, orthopedics, hepatology, and neurology [15,18]. Susanto et al. focused on the effects of ML-CDSSs in medicine, evidencing the lack of work conducted in PHC [23]. In addition, there is little high-quality evidence for improved performance or patient outcomes in clinical studies from other specialties [24].

This review focuses on the outcomes of AI-CDSSs implemented in PHC clinical settings. Given the diversity and novelty of the research literature about AI and AI-CDSSs in PHC, we decided to perform a scoping review. We followed the methodology described by Arksey and O’Malley [25]. Furthermore, we sought to answer the following questions:

1. How are AI-CDSSs being used in PHC?
2. How effective have AI-CDSSs been in PHC?
3. What are physicians’ perceptions toward them?

## 2. Methods

On 12 September 2023, we systematically searched 5 databases: PubMed, Scopus, CINAHL, IEEE, and Google Scholar. Only the first 110 papers were used for the latter to ensure that only the most relevant were screened. For a more comprehensive search, we screened the reference lists of the included studies and performed a manual search of related articles; we utilized the studies if relevant. 

We tailored our search string according to each database, and if applicable, we used a combination of Medical Subject Headings (MeSHs) and free text. An example of our search includes the following major topics connected with the Boolean Operator “AND”: 

Clinical Decision Support System: “clinical decision support system” OR “clinical decision system” OR “clinical decision support”. 

Artificial Intelligence: “artificial intelligence” OR “machine learning” OR “deep learning” OR “natural language processing” OR “neural network”. 

Primary Healthcare: “primary healthcare” OR “primary health care” OR “primary care physician” OR “general practitioner” OR “family physician” OR “community based” OR “community-based setting”.

We included articles regarding the use of CDSSs powered by AI in clinical PHC settings targeted to physicians. We limited our search to articles published between 2000 and 2023. Reasons for exclusion included articles about AI tools not in CDSSs, non-AI-based CDSSs, CDSSs directed to patient use, mobile apps, or non-PHC specialties. We excluded articles about the development and validation of AI-CDSSs using retrospective datasets.

**Figure 1:**
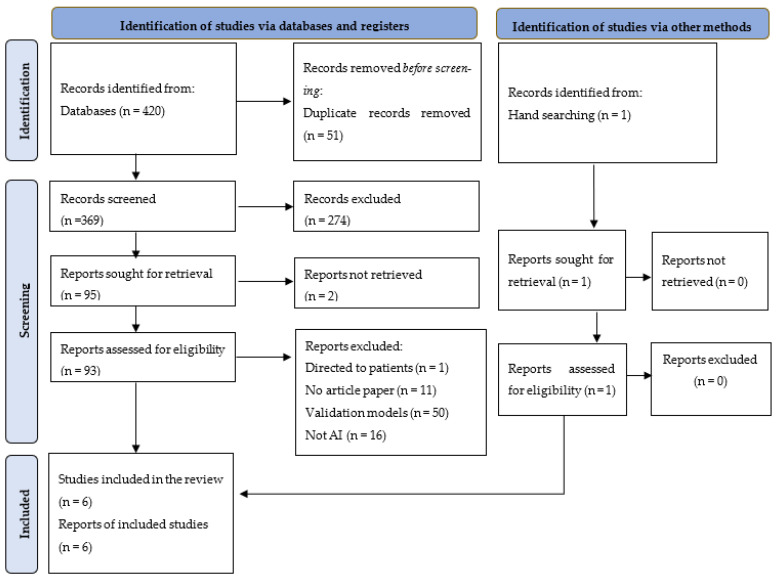
PRISMA flow diagram for the study-selection process.

We followed the Preferred Reporting Items for Systematic Reviews and Meta-Analyses Extension for Scoping Reviews (PRISMA-ScR) to ensure rigorous analysis (Figure 1) [26]. 

To guarantee a comprehensive analysis, we performed a meticulous search to extract information concerning country, study design, type of primary care setting, number of practices, patients and physicians involved, implementation time, study objective, CDSS task and outcome, type of AI leveraged, and user perception of the utilized CDSSs. A table was made from the pertinent information to compare results among articles.

## 3. Results

Our database search yielded 420 articles, and we found one more through manual search and reference list screening. References were imported and managed in EndNote 20. Identification for duplicate articles was performed manually and assisted by EndNote, resulting in the removal of 51 duplicate articles. After title and abstract screening, we sought 95 reports and retrieved 93. After the eligibility assessment, six eligible studies were identified and included in our analysis (Figure 1). 

### 3.1. Descriptive Analysis of the Studies

Of the six included studies, three are from the United States [27,28,29], while the rest are from the Netherlands [30], Spain [31], and China [32]. The two most common study designs were randomized clinical trials (two) [27,29], one of which was single-blinded [27], and observational cross-sectional studies (two) [28,32]. All six studies were performed in primary care clinics. The number of clinicians using the CDSSs was reported in four studies [28,29,31,32], and the number of patients assessed was reported in three studies [27,29,30]. 

### 3.2. Study Intentions

Four studies aimed to evaluate the performance of their CDSS [27,29,30,31], and two tried to analyze physicians’ attitudes and perceptions towards the CDSS [28,32]. Regardless of their intentions, all studies reported the effectiveness of the CDSS in performing its clinical task. 

### 3.3. CDSSs’ Characteristics and Applications

Four of six studies used ML for their CDSS [27,29,30,31], mainly natural language processing (NLP) [27,29,31], neural networks (NNs) [29], a Bayesian classifier [27], and DL [31]. The primary tasks of the CDSSs were diagnosis support [29,32], management recommendations [28,31], treatment recommendations [30], and complication prediction [27]. Secondary objectives included reducing physician burden for two studies [27,29] and reducing healthcare costs for another [27].

### 3.4. CDSSs’ Effectiveness

The AI-CDSSs in three studies accomplished their objective and improved physicians’ practice by enhancing diagnosis [29], treatment [30], and adherence to good-practice recommendations [31]. In two studies, the AI-CDSSs did not fulfill their objectives. Still, they achieved partial improvement by helping physicians inform their patients better [28], coordinating care, and reducing the time for chart reviewing [27]. The AI-CDSS of the remaining study was considered unfit by the physicians using it [32].

### 3.5. Physicians’ Experience with the CDSS

Interestingly, studies that primarily aim to assess the AI-CDSS’s performance obtained higher satisfaction levels. In Cruz et al., users described the system as fast, helpful, and unintrusive [31], while in Seol et al., physicians gave a median score of 7 on a 1–10 satisfaction scale [27]. Conversely, in Romero-Brufau et al., physicians reported they got less excited about AI and were more likely to feel it did not understand their job (*p* < 0.01), even though care was better coordinated (*p* < 0.01) and patients were better prepared to manage diabetes (*p* = 0.04) with the CDSS [28]. Additionally, in Wang et al., clinicians felt the CDSS was not optimized for their local context as it did not consider their patient load or resource limitations, resulting in limited utilization [32]. 

In Table 1 we depict a more thorough evaluation on the AI-CDSSs’ used, effectiveness, and outcomes. We also present additional results obtained from the studies. 

## 4. Discussion

Clinical Decision Support Systems aid physicians in tasks ranging from administrative automation and documentation to clinical management and patient safety [5]. They become more advantageous when integrated with EHRs as patients’ individual clinical profiles can be matched to the system’s knowledge base. This allows for customized recommendations and specific sets of administrative actions [8].

Regardless, clinician satisfaction remains low due to several factors, such as excessive time consumption, workflow interruption, suboptimal EHR integration, irrelevant recommendations, and poor user-friendliness [33,34]. A systematic review and meta-analysis by Meunier et al. found that many PCPs either perceived no need for CDSS assistance or disagreed with its recommendations [11]. Additionally, CDSSs disrupt physician workflow and increase their cognitive load, resulting in physicians spending more time to complete tasks and less time with patients [4]. Another significant concern is alert fatigue, forcing physicians to disregard up to 96% of the alerts offered by the CDSS, which sometimes may be detrimental to the patient’s well-being [3,5,9].

As the prevalence of chronic conditions continues to rise, the demand for healthcare services and documentation also increases, resulting in a higher volume of data usage. This incites a vicious cycle with EHRs and CDSSs overloading physicians and physicians entering incomplete, non-uniform data, leading to physician burnout and poor patient management [35,36]. In a study interviewing 1792 physicians (30% PCPs) about health information technology (HIT)-related burnout, 69.8% reported HIT-related stress, and 25.9% presented ≥1 symptom of burnout. Family medicine was the specialty with the highest prevalence of burnout symptoms and the third with the highest prevalence of HIT-related stress [37].

The overall burnout that primary physicians face represents one of the most significant challenges in PHC. Medication prescription errors are frequently reported among family physicians in the United States and other countries [38]. On top of that, approximately 5% of adult patients in the US experience diagnostic errors in the outpatient setting every year, with 33% leading to permanent severe injury or immediate or inevitable death [4].

In an attempt to diminish prescription errors, Herter et al. [30] implemented a system that considered patients’ characteristics to increase the proportion of successful UTI treatments and avoid overmedication and the risk of resistance. It increased the treatment success rate by 8% and improved adherence to treatment guidelines. While not yet implemented in PHC, one study in Israel reported the use of a CDSS powered by ML that identifies and intercepts potential medication prescription errors based on the analysis of historical EHRs and the patient’s current clinical environment and temporal circumstance. This AI-CDSS reduced prescription errors without causing alert fatigue [39].

The big data in EHRs may be a valuable tool for AI-CDSSs. By incorporating AI into CDSSs, they become more capable of clinical reasoning as they can handle more information and approach it more holistically. With ML, AI algorithms can identify patterns, trends, and correlations in EHRs that may not be apparent to physicians [15,16,19,40]. Likewise, they can learn from historical patient data to make predictions and recommendations for current patients [23,27].

In our study, the AI-CDSS in China was helpful for supporting physicians’ diagnoses and avoiding biases when in disagreement. Additionally, it provided similar cases to the current patient and relevant literature in real time. Physicians perceived this as a tool for training their knowledge, facilitating information research, and preventing adverse events [32]. In Yao et al. [29], the prediction capabilities of their AI-CDSS increased the diagnosis of low ejection fraction within 90 days of the intervention, achieving statistical significance. The intervention proved to be even more effective in the outpatient clinics.

With DL, AI arms CDSSs with the possibility of offering personalized treatment recommendations based on a patient’s unique medical history, genetics, and treatment responses [15,16,17,19,23,28,30,32,41,42]. Similarly, it can report abnormal tests or clinical results in real time and suggest alternative treatment options [23,29,31,32]. This immediateness can reduce the time needed for optimal treatment and increase physicians’ quality time spent with their patients [14,17].

We identified, as an example, the AI-CDSS used in Seol et al. [27], the Asthma-Guidance and Prediction System (A-GPS). Even though it did not prove a significant difference in its core objectives compared to the control, it reduced the time for follow-up care after asthma exacerbations and decreased healthcare costs. Additionally, it showed the potential to reduce clinicians’ burden by significantly reducing the median time for EHR review by 7.8 min.

**Figure 2:**
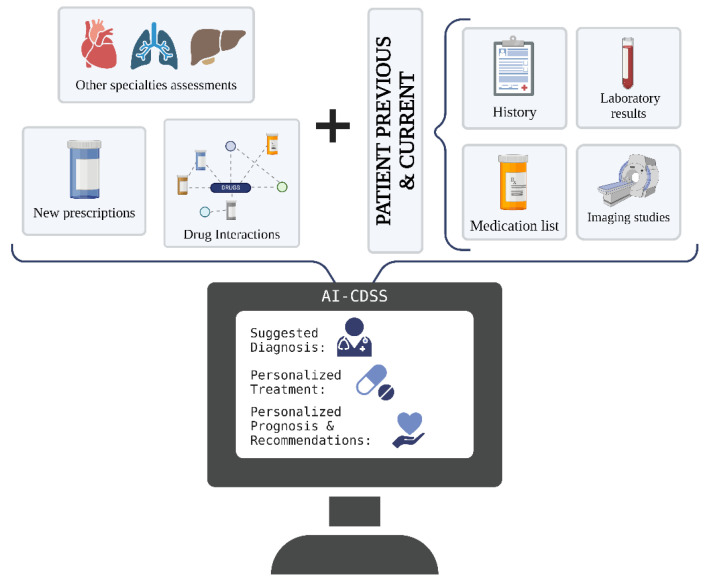
AI-CDSSs make patient-management processes smoother and more efficient, decreasing errors and misses while increasing productivity and personalized patient care. Created with BioRender.com [44].

When optimally developed, AI-CDSSs may be powerful tools in team-based care models, such as most PHC settings. They can assist physicians in delivering integrated services by organizing and ensuring that the entire patient-management process, from preventive care and coordination to full diagnostic workup, is effectively performed [13,43]. In addition, they can automate the process of note writing, extracting relevant clinical information from previous encounters and assembling it into appropriate places in the note [13,14,17]. This guarantees that physicians only focus on human interactions, which is the hallmark of primary care (Figure 2).

With their AI-CDSS, physicians in Cruz et al. improved their adherence to clinical pathways in 8 of the 18 recommendations related to common diseases in PHC; 3 were statistically significant [31]. Moreover, in Romero-Brufau et al., physicians perceived that the use of their AI-CDSS helped increase patients’ preparedness to manage diabetes and helped coordinate care [28].

Among our main findings is the scarcity of the literature research regarding AI-CDSS in PHC in real clinical settings, and not only the outcomes obtained but also the objectives of the studies, which were heterogeneous. Some focused on assessing the effectiveness of the systems [27,29,30,31], while others focused on the physicians’ attitudes toward them [28,32]. The effectiveness of the systems varied, with some proving to be more effective than their comparison group [29,30], some just proving to be somewhat useful [27,28,31], and others not being useful at all [32].

CDSSs and EHRs represent a burden for many physicians, leading to negative prejudices and biases toward them [4]. Additionally, there may be resistance and skepticism toward AI due to the increased workload that EHRs create [17]. Furthermore, there is mistrust in AI and concerns that AI may replace physicians [18,45].

Because of the latter, early research focuses on comparing and understanding physicians’ attitudes toward AI-CDSSs. This is the case of Romero-Brufau et al. [28] and Wang et al. [32]. In the former, the researchers found that physicians were less excited about AI and were more likely to feel like AI did not understand their jobs, even after becoming familiar with it. Clinicians gave a median score of 11 on a 1–100 scale, where 0 indicated that the system was not helpful. Only 14% of the physicians would recommend the AI-CDSS to another clinic, and only 10% thought that the AI-CDSS should continue to be integrated into their clinic within the EHR. Thirty-four percent believed the system had the potential to be helpful. This could be because the physicians perceived the interventions recommended by the system as inadequate, not sufficiently personalized for each patient, or simply unuseful [28].

In the same way, Wang et al. [32] reported that physicians felt the AI-CDSS “Brilliant Doctor” was not optimized for their local context, limiting or eliminating its use. Physicians reported that the confidence score of the diagnosis recommendations was too low, alerts were not useful, resource limitations were not considered, and it would take too long to complete what the system asked in order to obtain recommendations. These negative perceptions were not shared in N.P. Cruz et al. [31] and Seol et al. [27], where physicians were satisfied with the AI-CDSS. 

Even with AI proving actual improvement in several health fields, its general implementation faces some challenges. There are four major ethical challenges: informed consent for the use of personal data, safety and transparency, algorithmic fairness and biases, and data privacy [41,46]. First, most common AI systems lack explainability, what is known as the AI “black box.” This means that there is no way to be sure about which elements make the AI algorithm come to its conclusion. This lack of explainability also represents a main legal concern and reason why physicians distrust AI [47]. There is no consensus on to what extent patients should know about the AI that will be used, which biases it could have, or what risks it would pose. Moreover, what should be said about the incapacity to interpret the reason behind each recommendation fully? 

Secondly, for AI algorithms to function appropriately, they must be initially trained with an extensive dataset. For optimal training, at least 10 times the number of samples as parameters in the network are needed. This is unfeasible for PHC because of data and dataset scarcity, as most people do not have access to it [19,22]. On top of that, most healthcare organizations lack the data infrastructure to collect the data required to adequately train an algorithm tailored to the local population and practice patterns and to guarantee the absence of bias [6,15,35,48]. 

To solve this problem, some ML models are trained by using synthetic information, and others use datasets that may only derive from specific populations, leading to a selection bias [13,14,17,41,46,49]. The deficiency of real clinical backgrounds and racial diversity leads to inaccurate recommendations, false diagnoses, ineffective treatments, disparity perpetuation, and even fatalities [2]. Another phenomenon derived from data misalignment is the dataset shift, in which systems underperform due to small changes between the data used for training and the actual population in which the algorithm is being implemented [24,50,51].

This raises questions about accountability [16,41]. Who would be blamed in the case of an adverse event? Although there are forums and committees currently trying to settle this issue, right now it remains unclear, which leaves AI developers free of responsibility, physicians uncomfortable using it, and patients deprived of its potential benefits.

AI may have the capacity to grant equitable care among all types of populations, regardless of their socioeconomic backgrounds. However, the cost of implementing these technologies is high, and most developing countries do not have EHRs, or the ones they have are obsolete, sabotaging the implementation of efficient CDSSs [4,11]. This may partly explain why the success of CDSS in high-income countries cannot be translated to low-resource settings [6]. A reflection of the latter is the results in our paper, with five out of six AI-CDSS being tested in high-income countries. Additionally, the AI used in the “Brilliant Doctor” CDSS was not state-of-the-art nor optimally integrated into their EHR, making it difficult to work with [32]. 

Finally, the mistrust physicians and patients have towards AI is another critical challenge for its implementation [18]. In a study analyzing physicians’ perceptions of AI, physicians felt it would make their jobs less satisfying, and almost everyone feared they would be replaced. They also believed AI would be unable to automate clinical reasoning because AI is too rigid, and clinical reasoning is fundamentally the opposite. There were several other concerns, like the fear of unquestioningly following AI’s recommendations and the idea that AI would take control of their jobs [45].

In another study, the main reason for patients’ resistance to AI was the belief that AI is too inflexible and would be unable to consider their individual characteristics or circumstances [17]. There is also concern that increasing interaction, mainly with the AI-CDSS, would change the dynamics of the patient–provider relationship, rendering the practical clinic less accurate [14,21].

Recently, a vast effort has been put into the creation and implementation of explainable AI (XAI) models. These are described as “white-box” or “glass-box” models, which produce explainable results; however, they do not always achieve a state-of-the-art performance due to the simplicity of their algorithms [52,53]. To overcome this, there has been an increasing interest in developing XAI models and techniques to make the current models interpretable. Interpretability techniques, such as local interpretable model-agnostic explanations (LIMEs), Shapley Additive explanations (SHAPs), and Ancors, can be applied to any “black-box” model to make its output more transparent [52].

In healthcare, where the transparency of advice and therapeutic decisions is fundamental, approaches to explain the decisions of ML algorithms focus on visualizing the elements that contributed to each decision, such as heatmaps, which highlight the data that contributed the most to decision making [53]. Although XAI is not yet a well-established field, and few pipelines have been developed, the huge volume of studies on interpretability methods showcases the benefits that these models will bring to current AI utilization [52,53].

Making AI models more transparent will not eradicate mistrust by itself, as issues such as accountability and personal beliefs remain neglected. AI implementation should be a collaborative effort between AI users, developers, legislators, the public, and non-interested parties to ensure fairness [54]. More emphasis on conducting qualitative research testing the performance of AI systems would help physicians be sure their use is backed by sound research and not merely by expert opinion. AI education is paramount for a thorough understanding of AI models and, with this, more trust in using these models. With this in mind, some medical schools are upgrading their curriculums to include augmented medicine and improve digital health literacy [16]. Furthermore, some guidelines imply that trust can be achieved through transparency, education, reliability, and accountability [54].

The needs of both physicians and patients must be considered. According to Shortliffe and Sepulveda, there are six characteristics that an AI-CDSS must have to be accepted and integrated [55]:

- There should be transparency in the logic of the recommendation.
- It should be time-efficient and able to blend into the workflow.
- It should be intuitive and easy to learn.
- It should understand the individual characteristics of the setting in which it is implemented.
- It should be made clear that it is designed to inform and assist, not to replace.
- It should have rigorous, peer-reviewed scientific evidence.

To address some validation concerns and ensure transparent reporting, Vasey et al. proposed the DECIDE-AI reporting guideline, which focuses on the evaluation stage of AI-CDSS [24]. Additionally, there should be a specific contracting instrument to ensure that data sharing involves both necessary protection and fair retributions to healthcare organizations and their patients [35].

Co-development between developers and physicians is fundamental to obtaining adequate satisfaction levels and limitations for all parties [49]. Moreover, physicians need to stop thinking of AI as a replacement and instead start thinking of it as a complement. In PHC, AI and AI-CDSS could become pivotal points for improvement, mainly since reportedly half of the care provided can be safely performed by non-physicians and nurses [56]. Also, 77% of the time spent on preventative care and 47% on chronic care could be delegated to non-physicians [57]. With optimized AI-CDSS, the time dedicated to healthcare could change focus from quantity to quality. 

## 5. Limitations 

This is the first review to analyze the use and outcomes of AI-CDSS in real PHC clinical settings; however, there are two significant limitations. Firstly, while numerous studies with promising results exist regarding AI-CDSS in PHC, most of the algorithms presented are still in the validation phase or focusing on standardized patients. We decided to focus only on the papers documenting real clinical settings, which may not reflect the actual state of AI-based CDSSs in PHC. Secondly is the heterogeneity of the objectives among the included studies, with some searching for attitudes toward the AI-CDSS and not deepening into the system’s actual clinical outcomes. This prevents quantitative examinations of the results and may hinder the generalizability of the actual utility in the PHC setting. 

## 6. Recommendations for Future Research

Our review emphasizes the scarcity of research on AI-CDSSs in real PHC clinical settings. Due to its predictive capacities, AI has the potential to be a powerful tool for primary care, where promotive and preventive care is a priority. Conducting more clinical trials testing the performance of AI-CDSSs in PHC is paramount to prove their effectiveness. Furthermore, research focusing on the perception of primary physicians toward AI and AI-CDSSs is fundamental for developing user-centered systems. Ensuring the acceptability of these systems is crucial for enhancing their implementation. Increasing their use will expand the clinical information available to make better predictions, improve diagnostic and treatment capabilities, and decrease biases.

## 7. Conclusions 

AI-CDSSs have shown the potential to be advantageous in PHC’s core activities, assisting in diagnosis, patient management, and prevention. While there are still several challenges and limitations to their implementation, most research is focused on optimally overcoming them. Further work is needed to explore the broad spectrum of applications that the new AI-CDSSs have in several PHC real clinical settings and to measure their outcomes in clinical management, physicians’ work burden, and patient satisfaction and safety. 

## Figures and Tables

**Table 1:** Evaluation of included studies. Abbreviations: UTI (urinary tract infection); ML (machine learning); DL (deep learning); NLP (natural language processing); NN (neural network); CI (confidence interval); ECG (electrocardiogram); EF (ejection fraction); OR (odds ratio); HR (hazard ratio); A-GPS (Asthma-Guidance and Prediction System).

Author, Year	Country	Study Design	Primary Care Setting	Study’s Objective	Practices Involved	CDSS’s Objective	Implementation Duration	AI Model	Outcome	Barriers and Facilitators (Other Key Findings)
Cruz et al., 2019 [31]	Spain	Quasi-experimental	Primary care area of Castilla–La Mancha’s Health Service	Compare adherence to clinical pathways before and after implementation	24 centers: 86 physicians	Improve adherence to clinical pathways in real time	1 month	ML (DL, NLP)	Adherence improvement in 8 out of 18 recommendations. Statistically significant in three (*p* < 0.05)	1. It was the first measurement of the CDSS’s effectiveness 2. Average number of alerts per day per physician = 1.8
Romero-Brufau et al., 2020 [28]	USA	Observational cross-section	Regional primary care clinic	To explore attitudes toward AI before and after implementation among staff who used the AI-CDSS	3 clinics: 81 staff members (physicians, nurses, advanced practice providers, and clinical assistants)	1. Improve glycemic control in patients with diabetes 2. Identify patients at risk of poor glycemic control in the subsequent three months and provide tailored recommendations	3 months	N/A	1. Patients were better prepared to manage diabetes (*p* = 0.04) 2. Care was better coordinated (*p* < 0.01) 3. No improvement in the proportion of patients with adequate glycemic control	1. Outcomes are reported from the participants’ point of view 2. As survey participation was optional and anonymous, pre- and post-implementation response rates differed, and there was no individual pre–post-response pairing
Seol et al., 2021 [27]	USA	Randomized clinical trial	Primary care pediatric practices	Assess the effectiveness and efficiency of “A-GPS CDS” in optimizing asthma management	Single center: 184 patients (90 int., 94 ctrl.), children and families were blinded	1. Predict asthma exacerbation within 1 year 2. Reduce the clinician’s burden for reviewing and collecting clinical data from EHR to make a clinical decision 3. Reduce healthcare cost 4. Decrease time for follow-up care after asthma exacerbation	12 months	ML (Bayesian classifier and NLP)	1. No statistical difference for asthma exacerbation between intervention and control (OR: 0.82; 95% CI 0.34–1.96; *p* = 0.66) 2. 67% reduction in median time for chart review (3.5 min vs. 11.3 min; *p* < 0.001) 3. No significant difference in healthcare cost (*p* = 0.12) 4. No significant difference in follow-up care time after asthma exacerbation, though it was quicker (HR = 1.93; 95% CI: 0.82–1.45, *p* = 0.10)	1. Intervention was not synchronized with clinical visits but prescheduled, which might have reduced intervention effectiveness 2. The population was predominantly white (90%) and Scandinavian in ancestry. It may limit the generalizability of results
Wang et al., 2021 [32]	China	Observational cross-section	Rural first-tier clinic	Understand clinicians’ perception and usage of AI-CDSS in developing countries	6 clinics: 22 clinicians (physicians, surgeons, and Traditional Chinese Medicine practitioners)	1. Recommend diagnostic options 2. Suggest treatment and lab tests 3. Retrieve and show similar cases 4. Medical information search engine	6 months	N/A	There was limited or no use as clinicians felt the CDSS was not optimized for their local context	1. When used, clinicians found it helpful for: -Supporting their diagnosis -Facilitating information search -Training their knowledge -Preventing adverse events 2. The system’s algorithm did not utilize state-of-the-art AI techniques
Yao et al., 2021 [29]	USA	Randomized clinical trial	Primary care practices, community, and rural clinics	Assess whether an ECG-based CDT enables early diagnosis of low EF	45 clinics: 358 clinicians; 22,641 patients (11,573 int.; 11,068 ctrl.)	Early diagnosis of low ejection fraction	8 months	NN (NLP)	1. Increased diagnosis of low ejection fraction within 90 days of AI-ECG (1.6% in the control group vs. 2.1% in intervention. OR:1.32, CI: 1.08–1.61; *p* = 0.007) 2. Among patients with positive results, the intervention improved diagnosis from 14.5% (control) to 19.5% (intervention) (OR 1.43, CI: 1.08–1,91; *p* = 0.01) 3. Greater increase in diagnosis in those in outpatient clinics (1.0% control vs. 1.6% intervention, OR 1.71, CI: 1.23–2.37; *p* = 0.001)	1. Clinicians received alerts, reminders, and encouragement, which might give different outcomes in different practices 2. Nearly all patients had insurance coverage
Herter et al., 2022 [30]	Netherlands	Prospective Observational	Primary care practice	1. Compare the proportion of successful treatments before and during the study Success: no need for new tx after 28d post-initial tx 2. Determine the difference in prescribed antibiotics between tx vs. control and before vs. during implementation	36 intervention practices, 29 control: 1689 unique patients	Suggest treatment for patients with UTI	4 months	ML	1. 5% increase in successful treatment in the intervention group (z = 5.47; *p* < 0.001) 2. 8% increase in patients who use the software certainly (z = 4.95; *p* < 0.001) 3. 4% increase in the intervention group vs. control (z = 4.86; *p* < 0.001) 4. No significant difference in the proportion of high-tissue-penetration antibiotics before vs. during implementation	1. Only the results for females aged >70 were statistically significant (due to the sample size of the other subgroups) 2. Only 724 (61.1%) patients matched due to inconsistent CDSS use (before vs. after) 3. Only 724 (61.1%) patients matched due to inconsistent CDSS use (before vs. after)

## Data Availability

The raw data supporting the conclusions of this article will be made available by the authors upon request.
